# On the prediction of the phase distribution of bubbly flow in a horizontal pipe

**DOI:** 10.1016/j.cherd.2011.08.004

**Published:** 2012-01

**Authors:** G.H. Yeoh, Sherman C.P. Cheung, J.Y. Tu

**Affiliations:** a Australian Nuclear Science and Technology Organisation (ANSTO), PMB 1, Menai, NSW 2234, Australia; b School of Mechanical and Manufacturing Engineering, University of New South Wales, Sydney 2052, Australia; c School of Aerospace, Mechanical and Manufacturing Engineering, RMIT University, Victoria 3083, Australia

**Keywords:** Population balance, Computational fluid dynamics, Horizontal bubbly flow

## Abstract

Horizontal bubbly flow is widely encountered in various industrial systems because of its ability to provide large interfacial areas for heat and mass transfer. Nonetheless, this particular flow orientation has received less attention when compared to vertical bubbly flow. Owing to the strong influence due to buoyancy, the migration of dispersed bubbles towards the top wall of the horizontal pipe generally causes a highly asymmetrical internal phase distributions, which are not experienced in vertical bubbly flow. In this study, the internal phase distribution of air-water bubbly flow in a long horizontal pipe with an inner diameter of 50.3 mm has been predicted using the population balance model based on direct quadrature method of moments (DQMOM) and multiple-size group (MUSIG) model. The predicted local radial distributions of gas void fraction, liquid velocity and interfacial area concentration have been validated against the experimental data of Kocamustafaogullari and Huang (1994). In general, satisfactory agreements between predicted and measured results were achieved. The numerical results indicated that the gas void fraction and interfacial area concentration have a unique internal structure with a prevailing maximum peak near the top wall of the pipe due to buoyancy effect.

## Highlights

► DOMOM implementation for gas-liquid horizontal flow. ► Sensitivity study of non-drag force models under horizontal flow condition. ► Validation and comparison against MUSIG model and experimental data.

## Nomenclature

*a*: birth rate of population
*a*(*M*_*i*_,*M*_*j*_): coalescence rate in terms of mass
*A*: coefficient matrix
*b*: death rate of population
*b*(*M*_*i*_*,M*_*j*_): break-up rate in terms of mass
*a*_*if*_: interfacial area concentration
$B_{k}^{B},B_{k}^{C}$: mass birth rate due to break-up and coalescence
*C*: break-up model constant
*C*_*D*_: drag coefficient
*C*_*f*_: coefficient of surface area
*C*_*L*_: lift coefficient
*C*_*w*1_, *C*_*w*2_: wall lubrication coefficients
*C*_*TD*_: dispersion coefficient
*d*_*ij*_: equivalent diameter
*D*_*s*_: bubble Sauter mean diameter
$D_{k}^{B},D_{k}^{C}$: mass death rate due to break-up and coalescence
*f*: bubble size distribution
*f*_*BV*_: break-up volume fraction
*F*^lg^: total interfacial force
*F*_*B*_: break-up calibration factor
*F*_*C*_: coalescence calibration factor
$F_{\mathrm{drag}}^{\text{lg}}$: drag force
$F_{\mathrm{lift}}^{\text{lg}}$: lift force
$F_{\mathrm{lubrication}}^{\text{lg}}$: wall lubrication force
$F_{\mathrm{dispersion}}^{\text{lg}}$: turbulent dispersion force
*G*: production due to gravity
*h*_*o*_: initial film thickness
*h*_*f*_: critical film thickness
*j*: superficial velocity
*k*: turbulent kinetic energy
*m*^*k*^: moments of PSD
*M*: mass scale of gas phase (bubble)
*n*_*w*_: outward vector normal to the wall
*n*: number density
*N*: quadrature weight
*p*: pressure
*P*: shear production due to turbulence
*Re*_*b*_: bubble Reynolds number
*S*_*i*_: mass transfer rate due to coalescence and break-up
*S*_*k*_: moment source term
*u*: velocity vector
*u*_*t*_: turbulent velocity
*t*: physical time
*t*_*ij*_: time for two bubbles to coalesce
*v*: volume of bubble
*y*_*w*_: distance from the wall boundary

**Greek symbols**
*α*: void fraction
*α*_max_: maximum allowable void fraction
*β*: break-up kernel constant
*ɛ*: turbulence kinetic energy dissipation
λ: size of eddy in inertia sub-range
*λ*_min_: minimum size of eddy in inertia sub-range defined as $11.4{\left(\nu^{3}/\varepsilon \right)}^{1/4}$
*μ*: viscosity
*ρ*: density
*σ*: surface tension
*τ*_*ij*_: contact time for two bubbles
*ξ*: internal space vector of the PBE or size ratio between an eddy and a particle
*ζ*: quadrature abscissa
*Ψ*: additional variable

**Subscripts**
*e*: effective
*i*: index of abscissa of gas/liquid phase
*lam*: laminar
*tur*: turbulent
*td*: bubble-induced turbulence
*ts*: shear-induced turbulence

**Superscripts**
*g*: gas phase
*l*: liquid phase

## 1 Introduction

In many technological systems, gas–liquid flows are immensely important in the chemical and process industries. Among all the flow regimes that are prevalent in gas–liquid flows, bubbly flow conditions are of the greatest interest because of the capacity of handling processes requiring large scale interfacial areas for heat and mass transfer and efficient mixing processes.

Most computational studies of bubbly flows have thus far concentrated on vertical pipe configurations. In vertical flows, gravity mainly affects the axial gas-to-liquid relative velocity, but does not induce any lateral symmetry in either velocity or phase distribution. Nevertheless, in the case of horizontal (or even inclined) flows, the acceleration of gravity not only causes a significant flow asymmetry but also imposes an additional strong radial force. Thereby, under the combination of radial and axial forces, bubbles can travel neither vertically nor horizontally, which increases the difficulty in modelling horizontal bubbly flow in comparison to vertical bubbly flow. Also, the density stratification is often accompanied by a strong secondary flow.

Several measurement studies have been performed to describe the flow patterns in horizontal pipe flow. Govier and Aziz (1972) have classified the flow patterns into five groups, namely, bubbly, plug, slug, wave and annular. Taitel and Dukler (1976) and Weisman et al. (1979) have mapped these flow regimes in a two-dimensional coordinate system and predicted their transition for numerous fluid properties and pipe sizes. Nonetheless, many researchers have focused on the internal structure of horizontal bubbly flow. Some examples are Kocamustafaogullari and Wang (1991), Kocamustafaogullari and Huang (1994), Kocamustafaogullari et al. (1994), Andreussi et al. (1999), Iskandrani and Kojasoy (2001), Razzaque et al. (2003), Sanders et al. (2004) and Yang et al. (2004). Recently, interfacial structure of horizontal bubbly flow has been observed in 45-degree and 90-degree elbow by Kim et al. (2007, 2009).

Haoues et al. (2009) and Talley and Kim (2010) have developed a drift flux model to predict the integral flow characteristics of horizontal bubbly flow. Tselishcheva et al. (2010) applied the two-fluid model to simulate the void fraction and velocity profiles in a long straight horizontal pipe and a similar pipe with a 90-degree elbow. The local spatial two-phase geometrical internal structure (bubble diameter or interfacial area concentration) in such flow was found to be affected by the coalescence and break-up through the interactions among bubbles as well as between bubbles and turbulent eddies in turbulent flows. In order to predict the bubble size distribution, the population balance approach has been applied in order to accommodate the complicated bubble interaction mechanisms coupled with the two-fluid model. Ekambara et al. (2008) have applied the MUSIG model while Li et al. (2010) have adopted the Average Bubble Number Density (ABND) approach to investigate the internal phase distribution of a horizontal bubbly flow. For bubble-bubble and bubble-turbulence interactions, the major phenomenological mechanisms in bubbly flow conditions have been identified: (a) coalescence through random collision driven by turbulent eddies, (b) coalescence due to the acceleration of the following bubble in the wake of the preceding bubble, and (c) break-up due to the impact of turbulent eddies. Appropriate mechanistic models by Wu et al. (1998), Hibiki and Ishii (2002) and Yao and Morel (2004) for ABND and Prince and Blanch (1990), Chesters (1991), Luo and Svendsen (1996), Martinez-Bazan et al. (1999a,b), Lehr et al. (2002) and Lo and Zhang (2009) for multi-size bubble consideration have been established.

In the present of work, the method of moments (MOM) is adopted to predict the phase distribution of a horizontal bubbly flow. In contrast to the MUSIG model (Cheung et al., 2007a,b, 2008) which belongs to the class method, the bubble size distribution is tracked through its moments. The main advantage of this approach is essentially the computational economy that it entails. In contract to the MUSIG model where multiple classes (i.e. 10 classes or more) are normally required, the method condenses the problem by only tracking the evolution of a small number of moments (normally 4–6). This becomes ever crucial in the modelling of complex bubbly flows when the bubble dynamics is strongly coupled with already time-consuming calculations of turbulence multiphase flows. Owing to the difficulties experienced in expressing the transport equations in terms of the moments themselves, the DQMOM proposed by Marchisio and Fox (2005) has been applied. From the specific aspect in the modelling of bubbly flow, each node of the quadrature approximation is treated as a distinct gas phase. DQMOM, similar to MUSIG, can offer a powerful approach in describing bubbly flow undergoing coalescence and break-up processes in the context of computational fluid dynamic (CFD) simulations.

## 2 Model formulation

### 2.1 Two-fluid model

For isothermal horizontal bubbly flow, the equations for the ensemble-averaged of continuity and momentum governing each phase are solved simultaneously. By denoting the liquid as the continuous phase (*α*^*l*^) and the gas (i.e. bubbles) as disperse phase (*α*^*g*^), these equations can be written as:

*Continuity equation of liquid phase*

(1) $$\frac{\partial }{\partial t}(\rho^{l}\alpha^{l})+\nabla \cdot (\rho^{l}\alpha^{l}{\text{u}}^{l})=0$$

*Continuity equation of gas phase*

(2) $$\frac{\partial }{\partial t}(\rho^{g}\alpha^{g})+\nabla \cdot (\rho^{g}\alpha^{g}{\text{u}}^{g})=0$$

*Momentum equation of liquid phase*

(3) $$\frac{\partial }{\partial t}(\rho^{l}\alpha^{l}{\text{u}}^{l})+\nabla \cdot (\rho^{l}\alpha^{l}{\text{u}}^{l}{\text{u}}^{l})=-\alpha^{l}\nabla p+\alpha^{l}\rho^{l}\text{g}+\nabla \cdot [\alpha^{l}\mu_{e}^{l}(\nabla {\text{u}}^{l}+{\left(\nabla {\text{u}}^{l}\right)}^{T})+F^{\text{lg}}$$

*Momentum equation of gas phase*

(4) $$\frac{\partial }{\partial t}(\rho^{g}\alpha^{g}{\text{u}}^{g})+\nabla \cdot (\rho^{g}\alpha^{g}{\text{u}}^{g}{\text{u}}^{g})=-\alpha^{g}\nabla p+\alpha^{g}\rho^{g}\text{g}+\nabla \cdot [\alpha^{g}\mu_{e}^{g}(\nabla {\text{u}}^{g}+{\left(\nabla {\text{u}}^{g}\right)}^{T})]+F^{\mathrm{lg}}$$

The total interfacial force *F*^lg^ appearing in the liquid phase momentum equation (3) is formulated according to the appropriate consideration of different sub-forces affecting the interface between each phase. As demonstrated by Frank et al. (2004), the total interfacial force for the liquid phase is given by the drag, lift, wall lubrication and turbulent dispersion:

(5) $$F^{\mathrm{lg}}=F_{\mathrm{drag}}^{\mathrm{lg}}+F_{\mathrm{lift}}^{\mathrm{lg}}+F_{\mathrm{wall lubrication}}^{\mathrm{lg}}+F_{\mathrm{turbulent dispersion}}^{\mathrm{lg}}$$

Note that the total interfacial force in the gas phase momentum equation (4) is given by *F*^gl^ = −*F*^lg^. The inter-phase momentum transfer between gas and liquid due to the drag force can be obtained via the drag model of Ishii and Zuber (1979) as:

(6) $$F_{\mathrm{drag}}^{\mathrm{lg}}=\frac{1}{8}C_{D}a_{if}\rho^{l}|{\text{u}}^{g}-{\text{u}}^{l}|({\text{u}}^{g}-{\text{u}}^{l})$$

where *a*_*if*_ is the interfacial area concentration (=6*α*^*g*^/*D*_*s*_). The drag coefficient *C*_*D*_ in Eq. (6) has been correlated for several distinct Reynolds number regions for individual bubbles according to Ishii and Zuber (1979).

The non-drag forces are those of lift, wall lubrication and turbulent dispersion which these forces are directed perpendicular to the flow direction. Based on the formulation of Drew and Lahey (1979), the lift force in terms of the slip velocity and the curl of the liquid phase velocity can be described according to:

(7) $$F_{\mathrm{lift}}^{\mathrm{lg}}=\alpha^{g}\rho^{l}C_{L}({\text{u}}^{g}-{\text{u}}^{l})\times (\nabla \times {\text{u}}^{l})$$

Wall lubrication force as proposed by Antal et al. (1991), which acts in the normal direction away from the wall and decays with distance from the wall, can be expressed by:

(8) $$F_{\mathrm{wall lubrication}}^{\mathrm{lg}}=-\frac{\alpha^{g}\rho^{l}{\left[|{\text{u}}^{g}-{\text{u}}^{l}|(|{\text{u}}^{g}-{\text{u}}^{l}|\cdot {\text{n}}_{w}){\text{n}}_{w}\right]}^{2}}{D_{s}}\left(C_{w1}+C_{w2}\frac{D_{s}}{y_{w}}\right){\text{n}}_{w}$$

The turbulence induced dispersion developed by Lopez de Bertodano (1998) can be written in the form of:

(9) $$F_{\mathrm{turbulent dispersion}}^{\mathrm{lg}}=-C_{TD}\rho^{l}k^{l}\nabla \alpha_{l}$$

Alternatively, the turbulence induced dispersion based on the consistency of Favre-averaging developed by Burns et al. (2004) has also been applied, *viz.*,

(10) $$F_{\mathrm{turbulent dispersion}}^{\mathrm{lg}}=-{C^{\prime }}_{TD}\left[\frac{1}{8}C_{D}a_{if}\rho^{l}|{\text{u}}^{g}-{\text{u}}^{l}|\right]\frac{\mu_{tur}^{g}}{\rho^{g}Sc_{b}}\left(\frac{\nabla \alpha^{g}}{\alpha^{g}}-\frac{\nabla \alpha^{l}}{\alpha^{l}}\right)$$

In handling bubble induced turbulent flow, unlike single phase fluid flow problem, no standard turbulence model is tailored for bubbly flow. The standard *k* − *ɛ* model based on Launder and Spalding (1972) is considered for the continuous liquid phase with additional buoyancy turbulence source term being accounted within the transport equations. The governing equations for the turbulent kinetic energy *k* and turbulent dissipation *ɛ* are:

(11) $$\frac{\partial }{\partial t}(\rho^{l}\alpha^{l}k^{l})+\nabla \cdot (\rho^{l}\alpha^{l}{\text{u}}^{l}k^{l})=\nabla \cdot \left[\alpha^{l}\frac{\mu_{tur}^{l}}{\sigma_{k}}\nabla k^{l}\right]+\alpha^{l}(P-\rho^{l}\varepsilon^{l})$$

(12) $$\frac{\partial }{\partial t}(\rho^{l}\alpha^{l}\varepsilon^{l})+\nabla \cdot (\rho^{l}\alpha^{l}{\text{u}}^{l}\varepsilon^{l})=\nabla \cdot \left[\alpha^{l}\frac{\mu_{tur}^{l}}{\sigma_{\varepsilon }}\nabla \varepsilon^{l}\right]+\alpha^{l}\frac{\varepsilon^{l}}{k^{l}}(C_{\varepsilon 1}P-C_{\varepsilon 2}\rho^{l}\varepsilon^{l})$$

where *P* is the shear production defined by:

(13) $$P=\mu_{tur}^{l}\nabla {\text{u}}^{l}\cdot (\nabla {\text{u}}^{l}+{\left(\nabla {\text{u}}^{l}\right)}^{T})-\frac{2}{3}\nabla {\text{u}}^{l}\cdot (\mu_{tur}^{l}\nabla {\text{u}}^{l}-\rho^{l}{\text{u}}^{l})$$

The turbulent viscosity of the liquid phase is given by:

(14) $$\mu_{tur}^{l}=\mu_{ts}^{l}+\mu_{td}^{l}$$

where the shear-induced turbulence is given by

(15) $$\mu_{ts}^{l}=\frac{C_{\mu }\rho^{l}{\left(k^{l}\right)}^{2}}{\varepsilon^{l}}$$

while the bubble-induced turbulence which accounts for the effect of bubbles on liquid turbulence, based on Sato's bubble-induced turbulent viscosity model (Sato et al., 1981), can be expressed as

(16) $$\mu_{td}^{l}=C_{\mu p}\rho^{l}\alpha^{g}D_{s}|{\text{u}}^{g}-{\text{u}}^{l}|$$

The constants for the aforementioned *k* − *ɛ* model are: *C*_*μ*_ = 0.09, *σ*_*k*_ = 1.0, *σ*_*ɛ*_ = 1.3, *C*_*ɛ*1_ = 1.44, *C*_*ɛ*2_ = 1.92, and *C*_*μp*_ = 1.2. For the gas phase, the dispersed phase zero equation model is utilized; the turbulent viscosity of gas phase can otherwise be obtained as:

(17) $$\mu_{tur}^{g}=\frac{\rho^{g}}{\rho^{l}}\mu_{tur}^{l}$$

In Eqs. (3) and (4), the effective viscosity for the liquid and gas phases are subsequently given by:

(18) $$\mu_{e}^{l}=\mu_{lam}^{l}+\mu_{tur}^{l}$$

(19) $$\mu_{e}^{g}=\mu_{lam}^{g}+\mu_{tur}^{g}$$

### 2.2 Population balance models

For the purpose of predicting the Particle Size Distribution (PSD) within the bubbly flow, the population balance models based on the multiple-size group and quadrature approximation of the moment approaches are described below.

#### 2.2.1 DQMOM for bubbly flow

The moments of the PSD can be defined as

(20) $$m^{k}(\text{x},t)=\int_{0}^{\infty }f(\text{x},\xi ,t)\xi^{k}\;d\xi$$

In general, the first few moments provide important statistical descriptions on the population of particles which can be related directly to some physical quantities. One approach for computing the moment is to approximate the integrals in Eq. (20) using the numerical quadrature scheme – the quadrature method of moment (QMOM) as suggested by McGraw (1997). Instead of space transformation, the Gaussian quadrature closure is henceforth adopted to approximate the PSD according to a finite set of Dirac's delta functions. For bubbly flows, taking the bubble mass *M* as the preferred internal coordinate, the PSD takes the form:

(21) $$f(\text{x},M,t)\approx \sum_{i=1}^{n}N_{i}(\text{x},t)\delta (M-M_{i}(\text{x},t))$$

where *N*_*i*_ represents the number density or weight of the *i*th class and consists of all particles (bubbles) per unit volume with a pivot size or abscissa *M*_*i*_.

With the aim of solving multi-dimensional problems, Marchisio and Fox (2005) extended the MOM by developing the DQMOM where the quadrature abscissas and weights are formulated as transport equations. The main idea of the method is to keep track of the primitive variables appearing in the quadrature approximation, instead of the actual moments of the PSD. As a result, the evaluation of the abscissas and weights are obtained using matrix operations; the transport equations for weights and abscissas be derived after some mathematical manipulation according to:

(22) $$\frac{\partial N_{i}}{\partial t}+\nabla \cdot ({\text{V}}_{i}^{g}\;N_{i})=a_{i}$$

(23) $$\frac{\partial \zeta_{i}}{\partial t}+\nabla \cdot ({\text{V}}_{i}^{g}\;\zeta_{i})=b_{i}$$

where *ζ*_*i*_ = *N*_*i*_*M*_*i*_ is the weighted abscissas and the terms *a*_*i*_ and *b*_*i*_ are related to the birth and death rate of population which forms 2*n* linear equations where the unknowns can be evaluated via matrix inversion according to

(24) Aϕ=d

The 2*n* × 2*n* coefficient matrix *A* = [*A*_1_ *A*_2_] in Eq. (24) takes the form:

(25) $$A_{1}=\left[\begin{array}{ccc} 1 & \ldots & 1\\ 0 & \ldots & 0\\ -M_{1}^{2} & \ldots & -M_{n}^{2}\\ \vdots & \ddots & \vdots \\ 2(1-n)M_{1}^{2n-1} & \ldots & 2(1-n)x_{n}^{2n-1} \end{array}\right]$$

(26) $$A_{2}=\left[\begin{array}{ccc} 0 & \ldots & 0\\ 1 & \ldots & 1\\ 2M_{1} & \ldots & 2M_{n}\\ \vdots & \ddots & \vdots \\ (2n-1)M_{1}^{2n-2} & \cdots & (2n-1)M_{n}^{2n-2} \end{array}\right]$$

where the 2*n* vector of unknowns *ϕ* comprises essentially the terms *a*_*i*_ and *b*_*i*_: $\phi ={\left[a_{1}\ldots a_{n}\;b_{1}\ldots b_{n}\right]}^{T}=\left[\begin{array}{c} a\\ b \end{array}\right]$. In Eq. (24), the source or sink term is defined by *d* = [*S*_0_ … *S*_2*n*−1_]^*T*^. Applying the moment transformation, the *k*th moment term *S*_*k*_ is: $S_{k}(\text{x},t)=\int_{0}^{\infty }M^{k}S(\text{x},M,t)dM$. The sources and sinks of *S*(**x**, *M*, *t*) can be closed through the specification of constitutive relations.

In order to maintain consistency with the variables employed in the two-fluid model, the weights and abscissas can be related to the size fraction of the dispersed phase (*f*_*i*_) and an additional variable defined as *ψ*_*i*_ = *f*_*i*_/*M*_*i*_. Considering a homogeneous system in the present study in which the bubbles are assumed to travel with a common gas velocity $\left({\text{V}}_{i}^{g}\equiv {\text{u}}^{g}\right)$, the size fraction of *f*_*i*_ is thus related to the weights and abscissas by

(27) $$\rho^{g}\alpha^{g}f_{i}=N_{i}M_{i}=\zeta_{i}$$

Using the above expression and the variable *ψ*_*i*_, the transport equations become

(28) $$\frac{\partial }{\partial t}(\rho^{g}\alpha^{g}f_{i})+\nabla \cdot (\rho^{g}\alpha^{g}{\text{u}}^{g}f_{i})=b_{i}$$

(29) $$\frac{\partial }{\partial t}(\rho^{g}\alpha^{g}\psi_{i})+\nabla \cdot (\rho^{g}\alpha^{g}{\text{u}}^{g}\psi_{i})=a_{i}$$

The moment transform of the coalescence and break-up of the term *S*_*k*_ can be expressed as

(30) $$S_{k}=B_{k}^{C}-D_{k}^{C}+B_{k}^{B}-D_{k}^{B}$$

where

(31) $$B_{k}^{C}=\frac{1}{2}\sum_{i}\sum_{i}N_{i}N_{j}{\left(M_{i}+M_{j}\right)}^{k}a(M_{i},M_{j})$$

(32) $$D_{k}^{C}=\sum_{i}\sum_{j}M_{i}^{k}a(M_{i},M_{j})N_{i}N_{j}$$

(33) $$B_{k}^{C}=\sum_{i}\sum_{i}M_{i}^{k}r(M_{i},M_{j})N_{j}$$

(34) $$D_{k}^{B}=\sum_{i}M_{i}^{k}r_{i}N_{i}\;\text{with}\;r_{i}=\sum_{j}r(M_{i},M_{j})$$

where the terms *B* and *D* represent the birth and death rates of the coalescence and break-up of bubbles. From above, the weights *N*_*i*_ and *N*_*j*_ can be determined according to the definition given in Eq. (27).

#### 2.2.2 MUSIG model for bubbly flow

Instead of inferring the PSD to derivative variables (i.e. moments), the class method directly simulates its main characteristics using primitive variables (i.e. particle number density). Through this consideration, the continuous size range of particles is discretised into a series number of discrete size classes. For each class, a scalar (number density of particles) equation is solved to accommodate the population changes caused by intra/inter-group particle coalescence and breakage. The PSD is thereby approximated as:

(35) $$f(\text{x},M,t)\approx \sum_{i=1}^{n}N_{i}(\text{x},t)\delta (M-M_{i}(\text{x},t))$$

which incidentally is the same expression as proposed for the QMOM in Eq. (21). However, the groups (or abscissas) of class methods are now fixed and aligned continuously in the state space.

Assuming that each bubble class travel at the common velocity as in DQMOM, the number density equation Kumar and Ramkrishna (1996) can be expressed as

(36) $$\frac{\partial n_{i}}{\partial t}+\nabla \cdot ({\text{u}}^{g}n_{i})=B_{C}+B_{B}-D_{C}-D_{B}$$

In a form consistent with the variables used in two-fluid model, the transport equation in terms of size fraction becomes:

(37) $$\frac{\partial (\rho^{g}\alpha^{g}f_{i})}{\partial t}+\nabla \cdot (\rho^{g}\alpha^{g}{\text{u}}^{g}f_{i})=B_{C}+B_{B}-D_{C}-D_{B}$$

The birth and death rates can subsequently be written in terms of the size fraction according to:

(38) $$B_{C}={\left(\rho_{g}\alpha_{g}\right)}^{2}\frac{1}{2}\sum_{k}\sum_{l}f_{k}f_{l}\frac{M_{k}+M_{l}}{M_{k}M_{l}}a(M_{k},M_{l})$$

(39) $$D_{C}={\left(\rho_{g}\alpha_{g}\right)}^{2}\sum_{k}f_{i}f_{k}\frac{1}{M_{k}}a(M_{i},M_{k})$$

(40) $$B_{B}=\rho_{g}\alpha_{g}\sum_{k}r(M_{k},M_{i})f_{j}$$

(41) $$D_{B}=\rho_{g}\alpha_{g}f_{i}\sum_{k}r(M_{i},M_{k})$$

#### 2.2.3 Coalescence and break-up kernels for bubbly flow

According to Luo and Svendsen (1996), the bubble break-up rate can be modelled based on the assumption of bubble binary break-up under isotropic turbulence situation. Denoting the increase coefficient of surface area as $c_{f}=[f_{BV}^{2/3}+{\left(1-f_{BV}\right)}^{2/3}-1]$, where *f*_*BV*_ is stochastic break-up volume fraction of the daughter size distribution, the break-up rate in terms of mass can be obtained as

(42) $$b(M_{i},M_{j})=F_{B}C(1-\alpha^{g}){\left(\frac{\varepsilon^{l}}{d_{j}^{2}}\right)}^{1/3}\int_{\xi_{\text{min}}}^{1}\frac{{\left(1+\xi \right)}^{2}}{\xi^{11/3}}\times \text{exp}\left(-\frac{12c_{f}\sigma }{\beta \rho^{l}{\left(\varepsilon^{l}\right)}^{2/3}d^{5/3}\xi^{11/3}}\right)d\xi$$

where *ξ* = *λ*/*d*_*j*_ is the size ratio between an eddy and a particle in the inertial sub-range and consequently *ξ*_min_ = *λ*_min_/*d*_*j*_ and *C* and *β* are determined from fundamental consideration of drops or bubbles break-up in turbulent dispersion systems to be 0.923 and 2.0.

Bubble coalescence occurs via collision of two bubbles which may be caused by wake entrainment, turbulence random collision and buoyancy. In this present study, the turbulence random collision is only considered; all bubbles are assumed to be of spherical shape (i.e. wake entrainment becomes therefore negligible). The coalescence rate considering the turbulent collision taken from Prince and Blanch (1990) in terms of mass can be expressed as:

(43) $$a(M_{i},M_{j})=F_{C}\frac{\pi }{4}{\left[d_{i}+d_{j}\right]}^{2}{\left(u_{ti}^{2}+u_{tj}^{2}\right)}^{0.5}\text{exp}\left(-\frac{t_{ij}}{\tau_{ij}}\right)$$

where *τ*_*ij*_ is the contact time for two bubbles given by ${\left(d_{ij}/2\right)}^{2/3}/{\left(\varepsilon^{l}\right)}^{1/3}$ and *t*_*ij*_ is the time required for two bubbles to coalesce having diameters *d*_*i*_ and *d*_*j*_ estimated to be ${\left[{\left(d_{ij}/2\right)}^{3}\rho^{l}/16\sigma \right]}^{0.5}\text{ln}(h_{0}/h_{f})$. The equivalent diameter *d*_*ij*_ is calculated as suggested by Chesters and Hoffman (1982): *d*_*ij*_ = (2/*d*_*i*_ + 2/*d*_*j*_)^−1^. According to Prince and Blanch (1990), the initial film thickness *h*_*o*_ = 1 × 10^−4^ m and critical film thickness *h*_*f*_ = 1 × 10^−8^ m have been assumed. The turbulent velocity *u*_*t*_ in the inertial sub-range of isotropic turbulence (Rotta, 1972) which is given by: $u_{t}=\sqrt{2}{\left(\varepsilon_{l}\right)}^{1/3}{d_{i}}^{1/3}$. In Eqs. (42) and (43), *F*_*B*_ and *F*_*C*_ denote the calibration factors for break-up and coalescence.

## 3 Experimental details

**Fig. 1:**
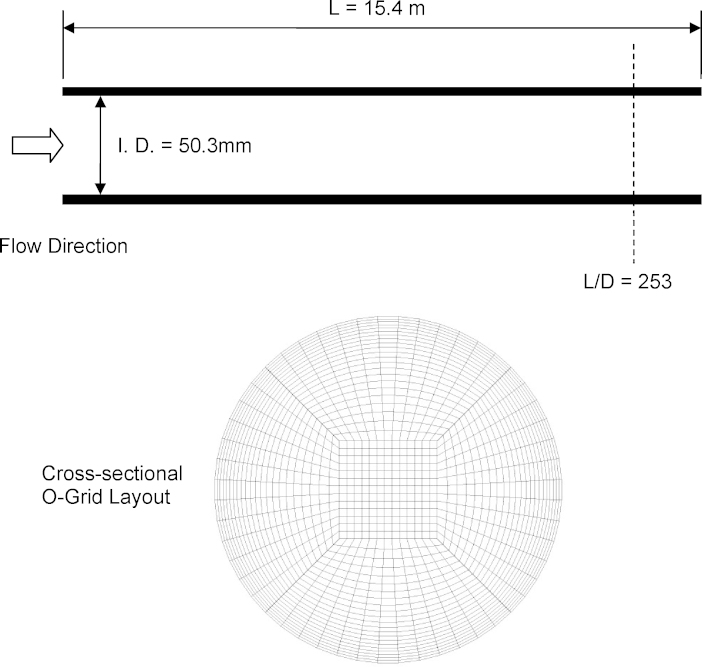
Schematic drawing of the experimental test section of Kocamustafaogullari and Huang (1994) and cross-sectional plane of the computational mesh.

The internal phase distributions of co-current air-water bubbly flow experiment have been carried out by Kocamustafaogullari and Huang (1994) in an inner diameter of 50.3 mm transparent horizontal pipeline of about 15.4 m in length such as depicted in the schematic drawing in Fig. 1. The temperature of the apparatus was kept at about 21–23 °C and the system pressure was about atmospheric. Local values of void fraction, interfacial area concentration, bubble passing frequency and axial velocity components were measured using the double-sensor resistivity probe technique. Axial development of bubbly flow structure was examined at 3 axial locations: *L*/*D* = 25, 148 and 253; the first measurement plane represented the entrance region when the flow was still developing while the second and third measuring planes depicted near fully developed flow pattern. Radial profiles were measured at 3 angles: *θ* = 0°, 45° and 90° where the angle *θ* was measured from the upper wall of the horizontal pipe orientation. A range of superficial liquid velocities 〈*j*_*f*_〉 and superficial gas velocities 〈*j*_*g*_〉 were performed. More details regarding the experimental set-up can be referred in Kocamustafaogullari and Huang (1994). The primary aim in this present study has been to assess the performance of DQMOM and compared against those of MUSIG in simulating the bubbly flow region at the nearly fully developed state of *L*/*D* = 253. Details of the flow conditions within the bubbly flow regime are summarized in Table 1.

## 4 Numerical details

For the two-fluid model, solutions to equations governing conservation of mass and momentum for each phase were obtained via the ANSYS Inc, CFX-5.7.1 computer code. For DQMOM, transport equations governing weights and abscissas were solved to predict the bubble size distribution of which the evaluation of the source terms *a*_*i*_ and *b*_*i*_ has been achieved through the utilization of a user subroutine incorporated within the CFD computer code. With regards to the coalescence and break-up kernels, calibration factors *F*_*B*_ and *F*_*C*_ have been adjusted to 0.15 and 0.05 for DQMOM as well as MUSIG model in this present study to match the experimental data. For comparison purpose, these dimensionless factors were kept constant for all flow conditions during the entire numerical calculations.

For DQMOM, four moments were adopted to explicitly track the distribution of bubble sizes ranging from 0 mm to 10 mm. For MUSIG model, 10 bubble classes were, however, equally divided for bubble sizes between 0 mm and 10 mm. For simplicity, all moments or bubbles classes in DQMOM and MUSIG model were assumed to travel in the same gas velocity which has been solved explicitly from the gas-phase of the two-fluid model.

Inlet conditions were assumed to be homogeneous with regards to the superficial liquid and gas velocities, void fractions for both phases and uniformly distributed bubble size in accordance with the flow conditions described in Table 1. At the pipe outlet, a relative average static pressure of zero was specified. A total number of 86,315 grid points was generated for the horizontal cylindrical pipe geometry. An O-grid layout was adopted for the distribution of grid points at the cross-sectional plane of the cylinder with more grid points being concentrated near the wall (see Fig. 1). A denser mesh of 128,765 grid points was tested. Nevertheless, the predicted cross-sectional averaged volume fractions between the two meshes yielded only differences of 2%. The mesh of 86,315 grid points was thereby employed to obtain the predicted results presented in the next section. Reliable convergence was achieved within 1500 iterations for a convergence criterion based on the RMS (Root Mean Square) residuals of 10^−4^ and a physical time scale of the fully implicit solution of 0.001 s

## 5 Results and discussion

### 5.1 Sensitivity of non-drag forces on time-averaged gas void fraction

In order to better understand the effect of different non-drag forces in horizontal bubbly flow, numerical calculations were carried out for three different cases using the DQMOM. The predicted void fraction distributions were compared against experimental data of Kocamustafaogullari and Huang (1994) at the dimensionless axial position *L*/*D* = 253 for 〈*j*_*g*_〉 = 0.419 m/s. For the reference case, the wall lubrication constants *C*_*w*1_ and *C*_*w*2_ were taken to have values of −0.01 and 0.05 as suggested by Antal et al. (1991). According to Ekambara et al. (2008), the lift and wall turbulent induced dispersion constants *C*_*L*_ and *C*_*TD*_ for horizontal pipe flow took on values of −0.2 and 0.5 respectively.

In the first case, simulations were performed by varying the lift coefficient while other non-drag forces were kept according to the reference case. The lift coefficient based on the formulation by Tomiyama (1998) has been prevalently utilized for vertical bubbly flow such has been demonstrated in Cheung et al. (2007a,b). The lift coefficient relationship takes the form:

(44) $$C_{L}=\left\{\begin{array}{ll} \text{min}\left[0.288\;\text{tanh}(0.121Re_{b}),f(Eo_{d})\right] & Eo<4\\ f(Eo_{d})=0.00105Eo_{d}^{3}-0.0159Eo_{d}^{2}-0.0204Eo_{d}+0.474 & 4\leq Eo\leq 10\\ -0.29 & Eo>10 \end{array}\right)$$

where *Eo* is the Eotvos number while the modified Eotvos number *Eo*_*d*_ is defined by $Eo_{d}=(g(\rho^{l}-\rho^{g})D_{H}^{2})/\sigma$ in which the maximum bubble horizontal dimension can be evaluated through the empirical correlation of Wellek et al. (1966): $D_{H}=D_{s}{\left(1+0.163Eo^{0.757}\right)}^{1/3}$. The above relationship allows positive and negative lift coefficients depending on the bubble size and also accounts for the effects of bubble deformation and asymmetric wake of the bubble. The predicted volume fraction profile for a constant *C*_*L*_ = −0.2 showed a maximum peak of 0.4 in the vicinity of the top wall and subsequently dropped below this peak at the top wall, which compared rather well with the measured profile. In contrast, the predicted volume fraction profile via the Tomiyama's correlation did not show the same volume fraction profile near the top wall and yielded a maximum value of about 0.9 at the top wall.

In the second case, simulations were carried out by varying the wall lubrication constants while other non-drag forces were kept according to the reference case. According to Krepper et al. (2005), the wall lubrication constants *C*_*w1*_ and *C*_*w2*_ have been adjusted to–0.0064 and 0.016 for vertical bubbly flow. This result showed that the volume fraction dropping only marginally below the maximum peak when compared to the reference case. Here again, better agreement was achieved through adopting the wall lubrication constants *C*_*w*1_ and *C*_*w*2_ of −0.01 and 0.05.

**Fig. 2:**
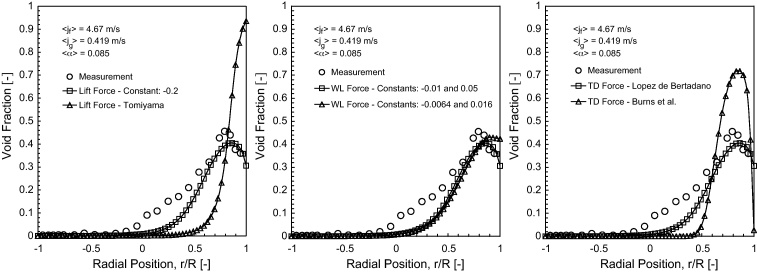
Effects of Lift Force, Wall Lubrication Force and Turbulent Induced Dispersion Force are assessed by comparing simulated and the experimental data of Kocamustafaogullari and Huang (1994) along the radial direction for *θ* = 0° at *L*/*D* = 253 of time-averaged void fraction distribution for superficial gas velocity of 0.419 m/s and superficial liquid velocity is 4.67 m/s: (a) effect of constant −0.2 and correlation by Tomiyama (1998); (b) effect of *C*_*w1*_ and *C*_*w2*_ of −0.0064 and 0.016 as proposed by Krepper et al. (2005) and *C*_*w1*_ and *C*_*w2*_ of −0.01 and 0.05; and (c) effect of turbulent induced dispersion force by Lopez de Bertodano (1998) and Burns et al. (2004).

In the third case, simulations were carried out by applying different turbulent induced dispersion forces while other non-drag forces were kept according to the reference case. For the turbulence induced dispersion force based on Burns et al. (2004), the constant ${C^{\prime }}_{TD}$ was assmued to be unity. It can be observed from Fig. 2(c) that the gas bubbles were found to be well dispersed for the turbulent induced dispersion force based on Lopez de Bertodano (1998). The turbulence induced dispersion force based on Burns et al. (2004) over predicted the maximum peak of the volume fraction by a considerable margin.

From these simulations, the lift coefficient based on Tomiyama's correlation, wall lubrication constants according to Krepper et al. (2005) and Favre-averaged turbulent induced force by Burns et al. (2004), typically applied for simulating vertical bubbly flow, did not show satisfactory agreement with the measured profile. However, the predicted profile by the reference case was found to give good match with the measured profile.

### 5.2 Time-averaged gas void fraction

**Fig. 3:**
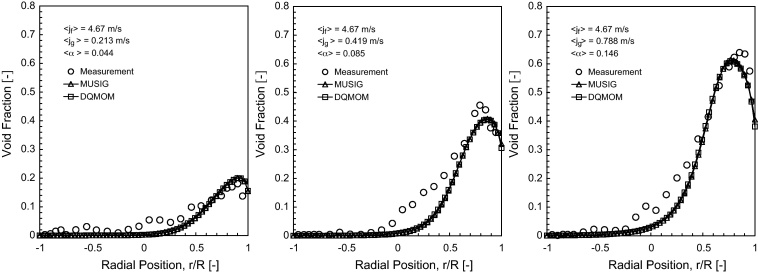
Local prediction of time-averaged void fraction distribution and experimental data of Kocamustafaogullari and Huang (1994) along the radial direction for *θ* = 0° at *L*/*D* = 253.

**Fig. 4:**
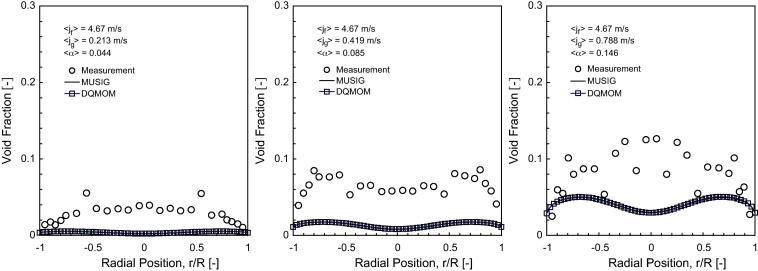
Local prediction of time-averaged void fraction distribution and experimental data of Kocamustafaogullari and Huang (1994) along the radial direction for *θ* = 90° at *L*/*D* = 253.

**Fig. 5:**
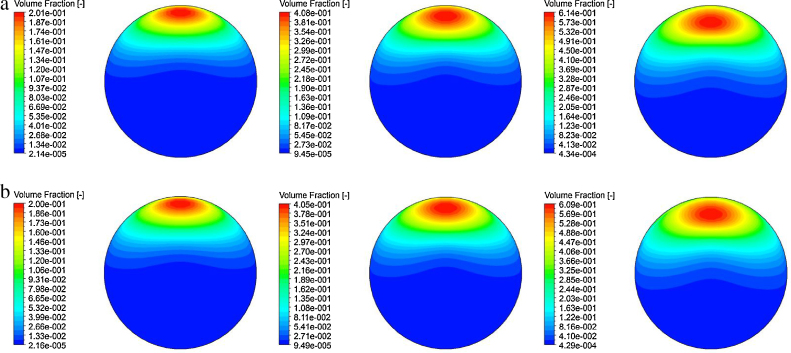
Cross-sectional contours of predicted time-averaged void fraction distribution at *L*/*D* = 253: (a) MUSIG and (b) DQMOM.

The predicted void fraction distribution of horizontal bubbly flow comparing against experimental data of Kocamustafaogullari and Huang (1994) for 〈*j*_*g*_〉 = 0.213 m/s, 〈*j*_*g*_〉 = 0.419 m/s and 〈*j*_*g*_〉 = 0.788 m/s at the dimensionless axial position *L*/*D* = 253 are shown in Figs. 3–5.

Satisfactory agreement was achieved between the measured and predicted radial profiles at *θ* = 0°. A peak persisted for the gas void fraction in the vicinity of the upper wall of the pipe for all the different superficial gas velocities. Physically, this can be explained by the upward migration of gas bubbles due to the upward buoyant force balancing with the downward wall lubrication force to prevent the gas bubbles from collapsing at the upper wall. In contrast to vertical bubbly flow, the movement of bubble towards the wall is generally caused by the balance between the opposing lift and wall lubrication forces. It can also be seen that the peak gas void fraction value increased from the superficial gas velocities from 〈*j*_*g*_〉 = 0.213 m/s to 〈*j*_*g*_〉 = 0.788 m/s to a level of about 0.6, which corresponded to the maximum packing condition of spherical solid particles. At this level, the maximum allowable void fraction has been exceeded indicating that gas bubbles have attained their saturation limit at the upper wall. At *θ* = 90°, the experimental radial profile of void fraction was shown to develop gradually from a saddle-type profile to a more parabolic profile with a single maximum at the pipe centre as the gas superficial velocity increased. Although the numerical prediction revealed similar increasing trend, the nature of a parabolic profile at 〈*j*_*g*_〉 = 0.788 m/s was not successfully captured by the current model. This discrepancy could be probably due to the insufficiency of the turbulence-induced force to dramatically push the bubbles away from the pipe wall or the specific requirement to further add a wall reaction force such as suggested by Tselishcheva et al. (2010) to counterbalance the buoyant force within horizontal bubbly flow; this wall reaction force remains to be fully tested and deserves separate thorough investigation in the future. In Fig. 4, the effect of buoyancy provoked the migration of gas bubbles towards the upper wall of the pipe was further evident with the void fraction distribution becoming highly asymmetrical in the pipe cross-section. Both MUSIG and DQMOM gave similar predictions albeit lower peak void fraction was obtained for the latter. The internal flow structure has a general similarity irrespective of the superficial gas velocities.

### 5.3 Time-averaged liquid velocity

**Fig. 6:**
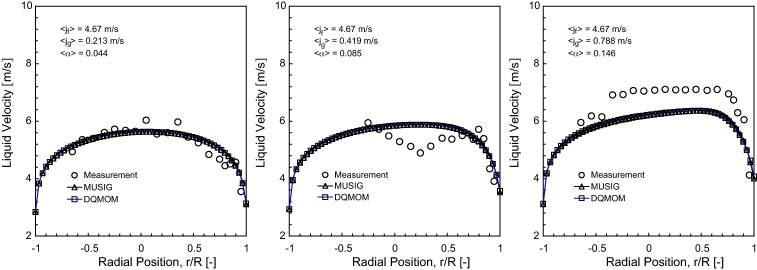
Local prediction of time-averaged liquid velocity distribution and experimental data of Kocamustafaogullari and Huang (1994) along the radial direction for *θ* = 0° at *L*/*D* = 253.

**Fig. 7:**
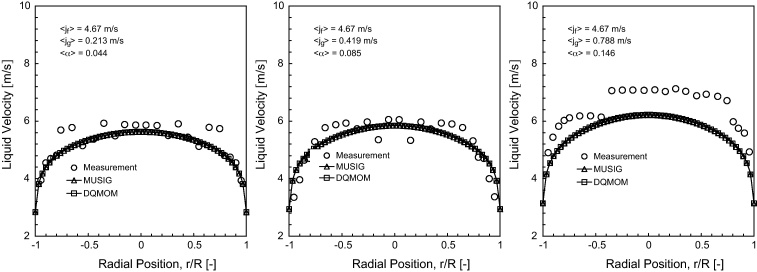
Local prediction of time-averaged liquid velocity distribution and experimental data of Kocamustafaogullari and Huang (1994) along the radial direction for *θ* = 90° at *L*/*D* = 253.

**Fig. 8:**
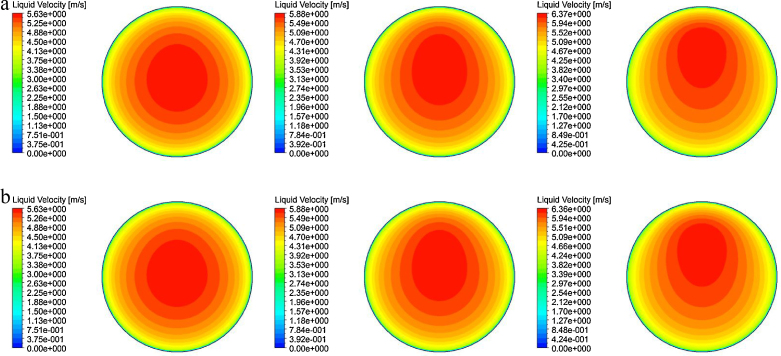
Cross-sectional contours of predicted time-averaged liquid velocity distribution at *L*/*D* = 253: (a) MUSIG and (b) DQMOM.

The predicted liquid velocity distribution of horizontal bubbly flow comparing against experimental data at *L*/*D* = 253 under the fixed superficial liquid velocity of 〈*j*_*f*_〉= 4.67 m/s are shown in Figs. 6–8.

Reasonably good agreement was achieved between the measured and predicted radial profiles at *θ* = 0° and *θ* = 90° except for 〈*j*_*g*_〉= 0.788 m/s. For this particular flow condition, the prevailing asymmetrical distribution was evidently observed which was attributed to the larger void fraction of gas in the upper wall of the pipe such as seen in Fig. 5. Based on the cross-section liquid velocity contours of the MUSIG and DQMOM predictions illustrated in Fig. 7, the increasing asymmetry of the liquid velocity distribution was also clearly observed with increasing gas superficial velocities. Of course, such observation would not be present in single liquid phase flow where the liquid velocity will move equally in the upper and bottom walls of the pipe, exhibiting a perfect axi-symmetry. On another note, the liquid velocities in bubbly flow system are, in general, greater than those in single phase flow system under the same flow conditions due to the inertial force acting between the gas and liquid phases. Since the liquid phase flow occupied a dominant position in the bottom section of the pipe, an interesting feature of the radial velocity profiles has been the close resemblance to a fully developed turbulent pipe flow irrespective of the superficial gas velocities.

Two significant observations can be made between the void fraction and liquid velocity distributions.

Firstly, although the experimentally measured void fraction profiles demonstrated large changes along the radial direction, the changes in the velocity profile shape were comparatively very small for different *θ*. No apparent peaks were observed in the liquid velocity profiles when compared to the void fraction profiles which showed a peak near the upper wall. According to Beattie (1972), there was no evidence to suggest a strong proportionate correspondence between the void fraction and velocity profiles. Similar phenomena have also been observed in the vertical bubbly flow performed by Hibiki and Ishii (2001) where the “wall peak” void fraction profile essentially has no influence on the velocity distribution.

Secondly, gas bubbles in vertical bubbly flow have a tendency to accelerate along the axial direction, which they are driven by the strong buoyant force. However, the buoyant force in horizontal bubbly flow, which now acts normal to the fluid flow, exerts a lesser contribution in pushing the gas bubbles to move along the axial direction than that in vertical bubbly flow. According to Kocamustafaogullari and Huang (1994) liquid velocities have been found to be only slightly greater than the velocities of the gas bubbles. The gas bubbles were accelerated by liquid inertia in a very short distance after injection but downstream of the bubbly flow, the local gas phase velocities followed closely to the local liquid phase velocities.

### 5.4 Time-averaged interfacial area concentration (IAC)

**Fig. 9:**
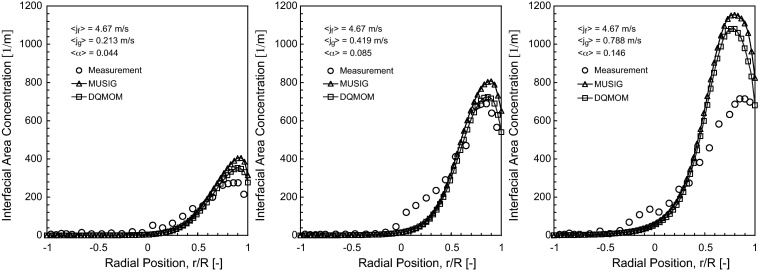
Local prediction of time-averaged IAC distribution and experimental data of Kocamustafaogullari and Huang (1994) along the radial direction for *θ* = 0° at *L*/*D* = 253.

**Fig. 10:**
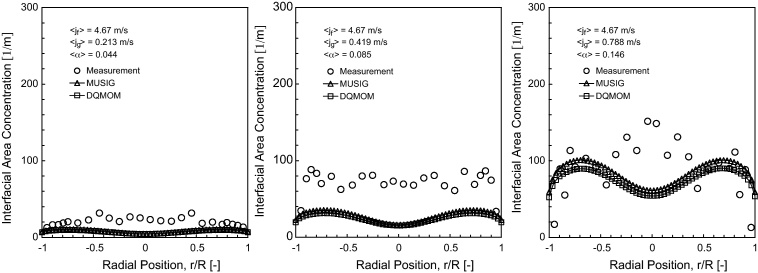
Local prediction of time-averaged IAC distribution and experimental data of Kocamustafaogullari and Huang (1994) along the radial direction for *θ* = 90° at *L*/*D* = 253.

**Fig. 11:**
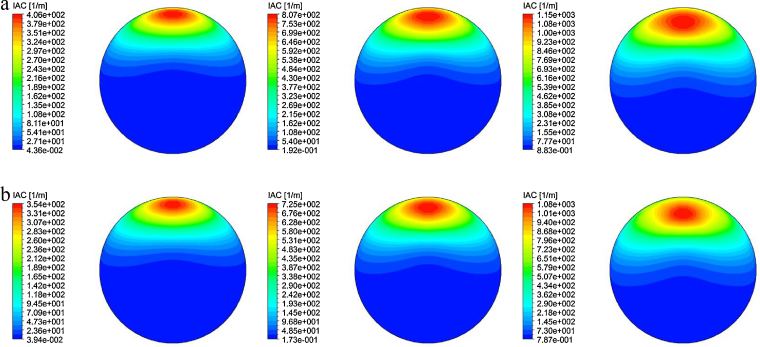
Cross-sectional contours of predicted time-averaged IAC distribution at *L*/*D* = 253: (a) MUSIG and (b) DQMOM.

The predicted interfacial area concentration (IAC) distribution of horizontal bubbly flow comparing against experimental data at *L*/*D* = 253 are shown in Figs. 9–11.

As observed in Kocamustafaogullari and Huang (1994), the Sauter mean bubble diameter distribution was nearly uniform for any given flow condition. It was therefore not surprising that the IAC followed very closely to the void fraction distributions. Here again, reasonably good agreement was achieved between the measured and predicted radial profiles at *θ* = 0° and *θ* = 90° except for 〈*j*_*g*_〉 = 0.788 m/s where significant over-prediction of the peak values were obtained, more so for MUSIG as compared to DQMOM. This was probably due to the lack of robustness of the population balance approach in predicting the bubble size being closed to the maximum packing limit which may require additional consideration of different bubble mechanistic models. Owing to the smaller Sauter mean diameter, higher than expected IAC was subsequently attained. In Fig. 10, similar to the void fraction distribution, the internal flow structure also showed a general similarity irrespective of the superficial gas velocities as seen from the cross-section contours of IAC.

## 6 Conclusion

A two-fluid model coupled with MUSIG and DQMOM has been presented in this paper to handle isothermal bubbly flow in a horizontal pipe. The evaluation of the source terms and the incorporation of appropriate set of equations for the abscissas and weights of DQMOM were implemented within ANSYS-CFX code to determine the temporal and spatial geometrical changes of the gas bubbles. In comparison to the local development of the void fraction, liquid velocity and interfacial area concentration, the DQMOM along with the two-fluid model was found to compare reasonably well with the measured values as well as those of the MUSIG model with the two-fluid model. This demonstrated the strong prospect for the possible application of the DQMOM in handling practical bubbly flow in a horizontal pipe.

## Figures and Tables

**Table 1:** Bubbly flow conditions and its inlet boundary conditions employed in the current investigation.

Superficial liquid velocity 〈*j*_*f*_〉 (m/s)	Superficial gas velocity 〈*j*_*g*_〉 (m/s)		
4.67 m/s	0.213 m/s	0.419 m/s	0.788 m/s
[*α*_*g*_|_*z*/*D*=0.0_ (%)]	[4.4]	[8.5]	[14.6]
[*D*_*S*_|_*z*/*D*=0.0_ (mm)]	[3.0]	[3.0]	[3.0]
